# Awareness, Cultural Beliefs, and Health-Seeking Behavior of Females in Cancer Screening: A Pilot Study in Rural South Africa

**DOI:** 10.3390/epidemiologia6040090

**Published:** 2025-12-10

**Authors:** Olufunmilayo Olukemi Akapo, Mojisola Clara Hosu, Mirabel Kah-Keh Nanjoh

**Affiliations:** 1 Department of Laboratory Medicine and Pathology, Faculty of Medicine and Health Sciences, Walter Sisulu University, Mthatha 5100, South Africa; mhosu@wsu.ac.za; 2 Department of Public Health, Faculty of Medicine and Health Sciences, Walter Sisulu University, Mthatha 5100, South Africa; mnanjoh@wsu.ac.za

**Keywords:** cervical cancer, cultural beliefs, knowledge, attitudes, screening, health-seeking

## Abstract

Background/Objectives: Cervical cancer is one of the most common cancers among women of reproductive age, with 80% of the cases occurring in developing countries. Cervical cancer is largely preventable by effective screening programs. This study assessed the knowledge, attitudes, cultural beliefs, and screening practices related to cervical cancer among women in the rural community of Lutubeni, Eastern Cape Province. Methods: A descriptive cross-sectional study was conducted among 95 women aged 25 years or older attending Lutubeni Clinic. Data was collected using a structured, validated questionnaire covering demographics, reproductive health, knowledge of cervical cancer, attitudes, cultural perceptions, and screening practices. Statistical analysis involved descriptive summaries, chi-square tests, and binary logistic regression. Results: Most participants exhibited poor knowledge of cervical cancer symptoms (47.4%) and risk factors (61.1%), with only 3.2% demonstrating good overall knowledge. Vaginal bleeding (60.0%) and foul-smelling discharge (50.5%) were the most recognized symptoms. Only 40.0% were aware of human papillomavirus (HPV) vaccination. While 87.4% knew about cervical cancer screening, only 55.8% had ever been screened. Of these, 43.2% had screened only once, primarily at the clinic (33.7%), mostly initiated by health professionals (41.1%). Positive attitudes toward screening were observed in 52.6%, while 88.4% held cultural beliefs that hindered open discussion about sexual health. Statistically significant factors associated with screening uptake included educational level (*p* = 0.047), knowledge of symptoms (*p* = 0.04), risk factors (*p* < 0.0001), prevention (*p* < 0.0001), treatment (*p* = 0.001), and attitudes (*p* < 0.0001). Independent predictors of poor screening practice were holding an associate degree (OR = 0.04, *p* = 0.042), having good preventive knowledge (OR = 0.02, *p* = 0.012), and having negative attitudes (OR = 36.22, *p* = 0.005). Conclusions: High awareness alone does not guarantee participation in cervical cancer screening in rural South Africa. Interventions must address cultural barriers, stigma, and negative perceptions while strengthening health education that links HPV vaccination with screening awareness. The unexpected association between associate degree attainment and poor screening underscores the complexity of behavioral determinants and warrants further investigation in larger cohorts.

## 1. Introduction

Cervical cancer is cancer affecting the cervix, an organ that connects the uterus and the vagina in women [1]. Globally, Cervical cancer is the fourth most common cancer in women, with around 660,000 incident cases and fatalities amounting to 350,000 in 2022, and accounts for 22% of all cancers in sub-Saharan Africa (SSA) [2,3,4]. It is the most common gynecological cancer worldwide, with the low- and middle-income countries bearing a disproportionate disease burden as a result of wide inequalities in human papillomavirus (HPV) vaccination exposure, socioeconomic factors, and access to preventive strategies [5]. Although cervical cancer is basically preventable through effective interventions, most women seek help late, when the disease has reached an advanced stage and is irreparable, eventually leading to death [6,7,8].

Global analysis from 185 countries found that the highest incidences were in Eastern, Western, and Southern African countries [9,10]. It further revealed that, except for Northern Africa, cervical cancer is the leading cause of cancer-related death in women in the African region. According to global mortality statistics, Africa bears the greatest burden, with a 24.55% mortality rate [10]. In SSA, the incidence and mortality rates of cervical cancer are 35 and 22.5 per 100,000 women annually, as compared to a lower figure of 6.6 and 2.5 per 100,000 women, respectively, in North America [11]. In SSA, 66% of cervical cancers are diagnosed at an advanced stage as compared to 25% in the UK, thus giving an insight into the disparity in age-standardized relative survival (ASRS) between SSA and high-income countries (HICs) [12]. The incidence and mortality rates of cervical cancer in South Africa are among the highest in SSA. Of the 117,316 incident cases and 76,745 fatalities recorded within the African region in 2020, 10,702 cases and 5870 associated deaths were reported from South Africa alone [13]. In contrast, a total of 4664 deaths due to female breast cancer were recorded in that same year, making cervical cancer the leading cause of cancer-related death among women in South Africa [13]. A study by the South African Medical Research Council reported cervical cancer as the most common cancer among females in the Eastern Cape, with a prevalence of 35% [14].

Persistent infection with high-risk human papillomavirus (hrHPV), particularly types 16 and 18, has been associated with over 70% of cervical cancers globally [4,10,15]. Although HPV infection leads to cervical cancer, other risk factors include HIV infection, oral contraceptives, early sexual escapades, multiple sexual associates, compromised immune system, and smoking [4,10,16]. Despite the magnitude of the disease, awareness is generally low globally and worse in LMICs [16]. Evidence suggests that increased awareness of signs, symptoms, and risk factors of cervical cancer leads to increased uptake of cervical screening, early detection, and subsequently leads to timely health-seeking behavior and prevention [17]. Being asymptomatic in its early stages, coupled with a long latent phase, screening is a highly essential preventative measure that detects precancerous cell changes on the cervical lining before progression to invasive cancer, thus reducing the burden of disease [15,16]. Although cytology-based pap smears remain the gold standard for screening to identify precursors, other methods include colposcopy and HPV-DNA testing as alternative screening methods, according to the 2017 South African guidelines [5,15,16]. Screening using a pap smear every 3–5 years with appropriate follow-up can lead to a reduced risk of cervical cancer by 80% [3,18].

Different factors, such as awareness of cervical cancer, level of education, and cultural and religious beliefs, influence women’s uptake of screening programs and services in developing nations. Culture and beliefs influence a person’s knowledge and perception of cancer screening, contributing to low participation in cervical screening programs and a crucial predictor of health-seeking behavior [3,6]. Cultural diversity and socio-economic factors are attitudinal barriers to women’s access to cervical screening services [19]. Attitudes play a crucial role in shaping health-seeking behavior. A negative attitude is associated with low uptake of screening services for the prevention and treatment of cervical cancer among women in SSA [19]. Hence, the study aimed to assess the knowledge, attitudes, and cultural beliefs of women regarding cervical cancer screening who attended primary healthcare centers in a rural community in the Eastern Cape.

## 2. Materials and Methods

### 2.1. Study Design, Setting, and Population

This community-based, descriptive, cross-sectional study was conducted at the Lutubeni Clinic, a primary healthcare facility located in the rural community of Mqanduli, Oliver Tambo District, Eastern Cape Province, South Africa. The clinic serves residents of Lutubeni and the surrounding villages of Mampingeni, Mandqobe, Gomatana, Haji, Khawula, and Mbozisa. The target population consisted of women aged 25 years and older who had resided in Lutubeni for at least one year. The study population consisted of female study participants who attended the clinic during the data collection period. Eligible participants were recruited using convenience sampling based on their availability and willingness to participate. This design was chosen because it is suitable for assessing knowledge, attitudes, cultural beliefs, and screening practices at a single point in time, allowing for the identification of associations between variables within a community setting. Given the exploratory nature of this pilot study and the resource constraints, a cross-sectional design is the most feasible approach, providing valuable baseline data to inform future research.

#### Eligibility Criteria: Inclusion and Exclusion Criteria

Study participants aged 25 years and older who resided in Lutubeni for at least one year and who provided informed consent were eligible for inclusion. Males, critically ill individuals, eligible participants who did not consent to participate, and individuals who decided they no longer wanted to participate in the study were excluded from the study.

### 2.2. Sample Size Calculation

The sample size was calculated using the formula: n = *Z*^2^α × *p*(1 − *p*)/*e*^2^. Where *Z* = 1.96 for a 95% confidence interval, *p* = 0.355 (estimated cervical screening prevalence in South Africa) [20], and *e* = 0.10 (margin of error). This margin was chosen due to the exploratory nature of the pilot study, the rural setting with limited resources, and anticipated challenges in participant recruitment. Narrower margins (e.g., 5%) are ideal in large-scale public health studies; a 10% margin was deemed acceptable to balance feasibility with statistical validity in this context. The estimated sample was 88 participants; accounting for a 15% non-response rate, the final target was 102 participants.

### 2.3. Data Collection, Management, and Analysis

Data was collected over four weeks (5–26 October 2022) using a validated structured questionnaire comprising five sections: (A) sociodemographic characteristics, (B) reproductive and lifestyle factors, (C) knowledge about cervical cancer and screening, (D) attitudes toward screening, and (E) cultural beliefs. The instrument was developed from previously published studies [21,22], translated into isiXhosa, and then retranslated into English for accuracy. Data collection was conducted by nine trained medical students from Walter Sisulu University during Community-Based Education and Service (COBES) rotations.

#### 2.3.1. Validity and Reliability

Face validity was established through review by academic supervisors to confirm the clarity and appropriateness of the content. Departmental reviewers assessed content validity to ensure completeness and coverage. Reliability was maintained through consistent application of the structured worksheet and cross-checking of extracted data to verify internal consistency and accuracy.

#### 2.3.2. Ethical Considerations

Ethical clearance was obtained from the Walter Sisulu University Health Sciences Research Ethics Committee (protocol code 086/2022; approval date 12 September 2022). Administrative approval was also obtained from the Lutubeni clinic management and the Eastern Cape Department of Health. Written informed consent was obtained from all participants prior to data collection, and participation was voluntary with the option to withdraw at any time without consequence. To maintain confidentiality, no identifying information was collected, and all data was anonymized and stored securely with access limited to the research team.

### 2.4. Statistical Analysis

Data was entered into Microsoft Excel and analyzed using SPSS version 27 (IBM Corp., Armonk, NY, USA). Continuous variables were summarized as means ± standard deviations or medians with interquartile ranges (IQR), depending on data distribution. Categorical variables were described as frequencies and percentages. Associations between sociodemographic, knowledge, and attitude variables and screening practice were tested using the Chi-square or Fisher’s exact test. Statistically significant variables (*p* < 0.05) were entered into a binary logistic regression model, as the dependent variable (screening practice) was separated into ‘good’ and ‘poor’. This approach was used to identify independent predictors of screening practice. Knowledge Scoring: Correct answers received 1 point; incorrect answers received 0. Composite knowledge scores were calculated and categorized using Modified Bloom’s criteria: good (80–100%), moderate (50–79%), and poor (<50%). Attitude Scoring: Attitudes were assessed using a 5-point Likert scale. Total scores were calculated, and participants were dichotomized into positive (<mean score) and negative (≥mean score) attitude categories. The mean score was used as a threshold to separate attitudes into positive (<mean) and negative (≥mean), a method commonly applied in similar community-based studies where no standardized cut-off exists. This approach allowed us to categorize participants relative to the overall distribution of responses within the study population, ensuring context-specific interpretation of attitudes [20]. Screening Practice: Defined as “good” if participants had ever undergone cervical cancer screening; otherwise, ‘poor. Missing data, questionnaires lacking critical information (such as age or cervical screening status) were excluded from the final analysis. For variables with minimal missing responses, such as marital status, the number of missing cases was indicated in the results.

## 3. Results

A total of 98 study participants aged 25 years and older participated in the study, yielding a response rate of 96.1%. After excluding three questionnaires due to missing critical data (age and cervical screening status), the final sample comprised 95 participants, which exceeded the minimum required sample size (n = 88). Only marital status had incomplete responses (n = 4 missing).

The results presented below include analyses of participants’ sociodemographic, reproductive, and lifestyle characteristics, as well as their knowledge, attitudes, and cultural beliefs regarding cervical cancer and screening. Factors influencing cervical cancer screening practices among women in Lutubeni were also assessed.

### 3.1. Sociodemographic Characteristics of the Study Population

The study sample consisted of 95 women aged 25–68 years (median: 39; IQR: 30–48), with the largest proportion (43.2%) falling within the 25–35 years age range. The majority self-identified as Christian (94.7%), while smaller proportions reported African spirituality (3.2%) or Zionist beliefs (2.1%). Most participants were either single (42.1%) or married (41.1%). Educational levels varied, with 42.1% having completed secondary education, and 11.6% possessing associate degree or degree qualifications. Almost one-third (30.5%) of participants were unemployed, 26.3% were housewives, while about one-quarter of participants were government employees (25.3%). Monthly income was predominantly below 2000 Rands (South African currency) (60%), and nearly all respondents (98.9%) identified as heterosexual (Table 1).

### 3.2. Reproductive Characteristics of Study Participants Residing in Lutubeni

The reproductive characteristics of 95 women residing in Lutubeni revealed that almost all participants (98.9%) had previously engaged in sexual activity, with the median age at sexual debut being 18 years (IQR: 16–21; range: 10–30 years). Over half (51.6%) reported initiating sexual activity between ages 15 and 18, while 7.4% reported initiation before age 15 (Table 2). A history of casual sexual partnerships was reported by 45.3% of participants, whereas 53.7% reported having only steady partners. Modern contraceptive use was high (78.9%), with a median usage duration of 7 years (IQR: 5–10). The majority (40.0%) had used contraceptives for 5–9 years, 21.1% for ≥10 years, and 16.8% for less than 5 years. Only 2.1% reported a maternal history of cervical cancer. Condom use was reported by 75.8% of participants. A small proportion (12.6%) were current cigarette smokers, and 46.3% reported alcohol consumption.

### 3.3. Knowledge Level About Cervical Cancer and Screening Among the Participants

Out of the 95 participants, quite a number demonstrated poor knowledge of cervical cancer symptoms (47.4%), with the majority having poor knowledge of risk factors (61.1%) (Table 3). Nearly a quarter (24.2%) reported good knowledge of symptoms, with vaginal bleeding (60%) and foul-smelling vaginal discharge (50.5%) being the most recognized symptoms, while pain during intercourse was also reported, but in a lower proportion (45.3%). Regarding risk factors of cervical cancer, 47.4% acknowledged multiple sexual partners as a risk, fewer participants recognized associations with HPV infection (24.2%), while 30.5% and 41.1% of participants recognized early sexual debut and smoking as risk factors, respectively. Overall, 32.6% of participants reported having no knowledge of any risk factors. Slightly more than half (54.7%) had poor knowledge, and only 40% were aware of HPV vaccination as a preventive measure against cervical cancer. Awareness of cervical cancer screening was 87.4%, demonstrating good knowledge, and 90.5% acknowledged the existence of screening programs. Overall, the composite knowledge score revealed that 46.3% of participants had poor knowledge, 50.5% had moderate knowledge, and only 3.2% demonstrated good knowledge regarding cervical cancer and its screening.

### 3.4. Overall Knowledge Level and Age Group of the 95 Participants from the Lutubeni Community

**Figure 1:**
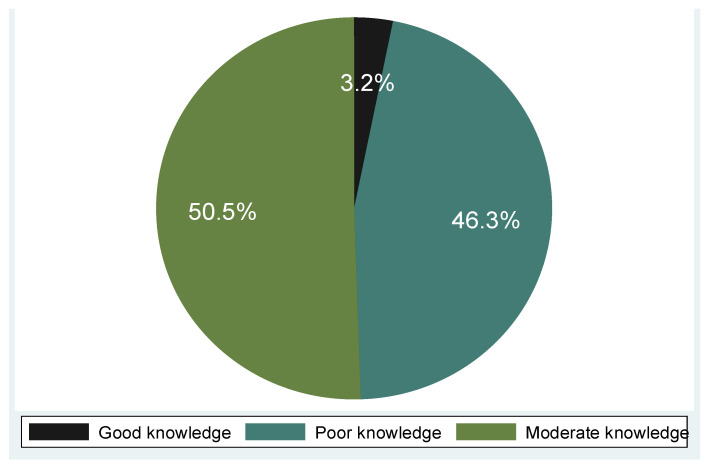
Overall knowledge level of the 95 participants from the Lutubeni community.

**Figure 2:**
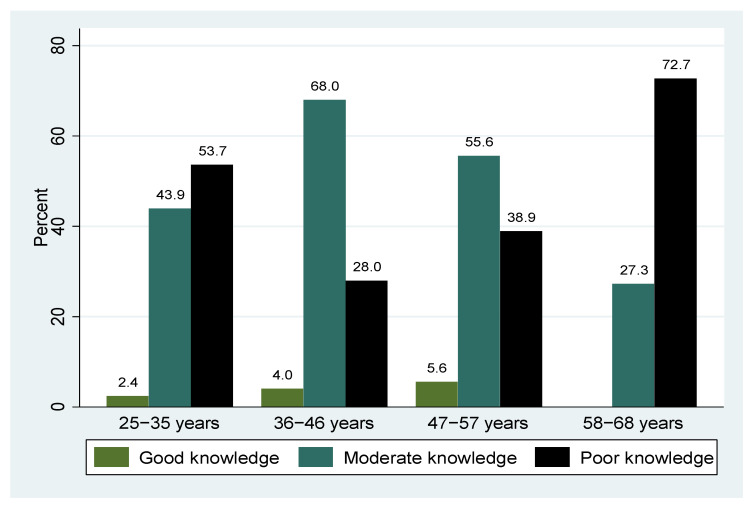
Age group by overall level of knowledge.

Approximately half of the participants (50.5%) had moderate knowledge of cervical cancer (Figure 1). Poor knowledge was highest among participants aged 58 to 68 years, moderate among those in the 36- to 46-year age group, and good knowledge among those in the 47- to 57-year age group (Figure 2).

Coverage refers to the proportion of women who have ever been screened within each age group. While the 36–46 age group meets the WHO benchmark, uptake is substantially lower among younger women (25–35 years) and declines again after midlife, indicating gaps in consistent lifetime screening(Table 4).

### 3.5. Attitude of the Participants Towards Cervical Cancer Screening

Analysis of the participants’ attitudes is presented in Table 5. From all the participants, more than two-thirds (67.4%) strongly agreed that cervical cancer is becoming a problem in South Africa, while 40.0% of the participants agreed that it is the leading cause of cancer-related death among women in South Africa. Just over 40% (41/95, 43.2%) of the participants agreed that any adult woman can acquire cervical cancer, and 43.2% (44/95) agreed that cervical cancer cannot be transmitted from one person to another. Most (50.5%) of them agreed that screening helps in the prevention of cervical cancer, while a moderate (38.9%) agreed that screening causes harm.

### 3.6. Overall Attitudes Scores and Age-Stratified Attitudes of Participants

**Figure 3:**
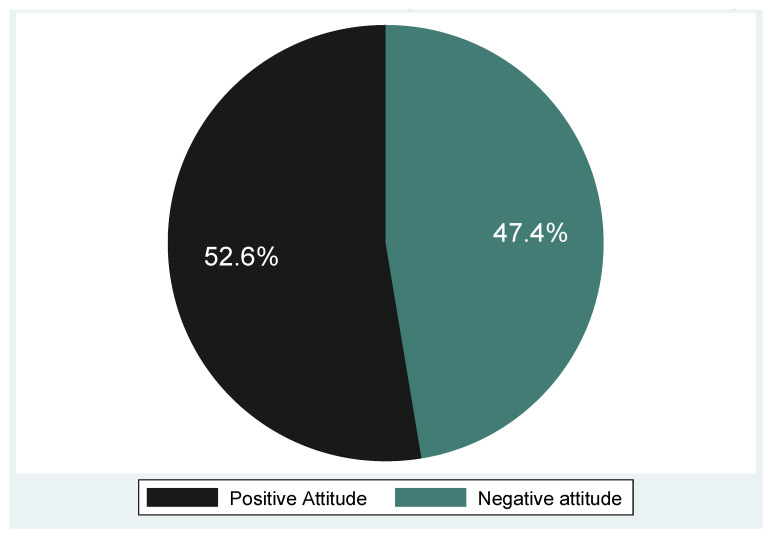
Attitude of the participants towards cervical cancer and screening.

**Figure 4:**
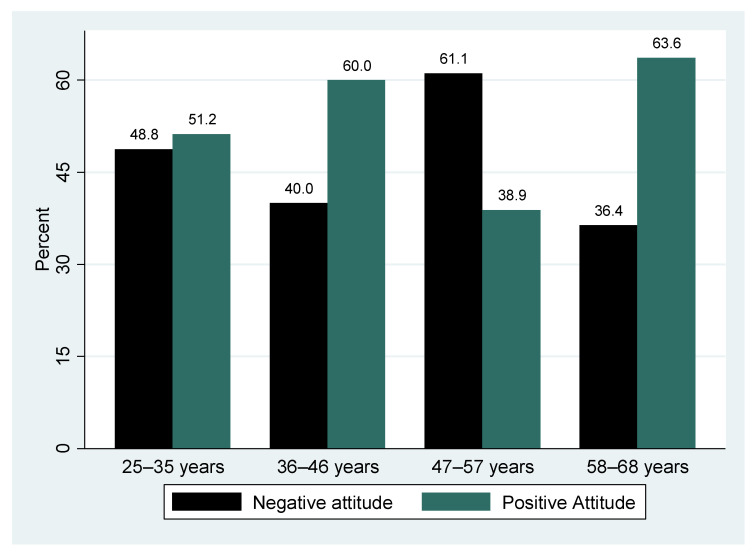
Age group stratified by attitude toward cervical cancer and screening.

The results of the overall attitude score indicate that slightly more than half (52.6%, n = 50) of the participants had a positive attitude towards cervical cancer and screening, while almost half (47.4%, n = 45) had a negative attitude (Figure 3). Figure 4 shows that positive attitude was highest among the elderly (58–68 years), while the negative attitude was highest among the 47–57 year age group (Figure 4). The differences in the proportion of positive and negative attitudes across the different age groups was not statistically significant (*p* = 0.480).

### 3.7. Cultural Views Regarding Cervical Cancer Among Study Participants

Study participants with Cultural views regarding cervical cancer and cervical cancer screening perceived that these aspects were false (Table 6).

### 3.8. Cervical Screening Practice and Cervical Screening Experiences of the Participants

**Figure 5:**
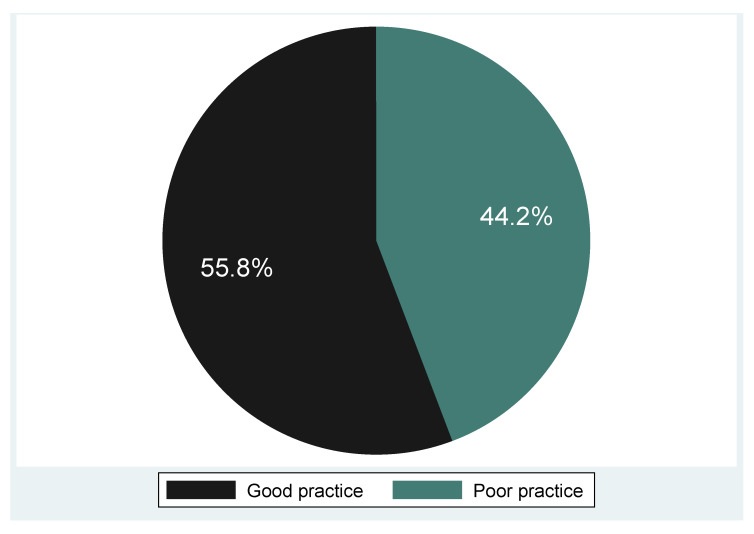
Cervical screening experiences of the participants.

The majority (55.8%) had screened for cervical cancer, and 42 (44.2%) had never screened for cervical cancer (Table 7). Among those who had screened, 43.2% reported screening only once. A lower proportion (33.7%) of them were screened in the clinic with (44.1%) of the participants screened at the initiation of a health professional, (13.7%) were self-initiated and a minute proportion (1.1%) were initiated by a friend. Eighty-eight (92.6% n = 88) participants had screened for other reproductive issues such as HIV and STIs. Based on screening experiences, 55.8 percent were labeled as having good screening practice and 44.2 percent as poor screening practice (Figure 5).

### 3.9. Age Group by Cervical Cancer Screening Practice

**Figure 6:**
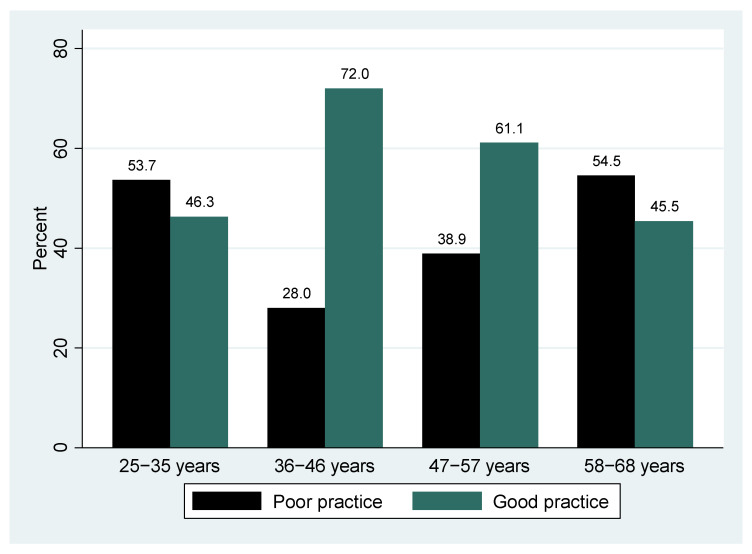
Age Group by Cervical Cancer Screening Practice.

The proportion of participants who screened for cervical cancer across the different age groups (Figure 4, Figure 5 and Figure 6) was not statistically significantly different (*p* > 0.05), although good practice was highest among the middle age group (36–46 years) and poor practice among the elderly (58–68 years).

### 3.10. Factors Associated with Practice Towards Cervical Cancer Screening

Bivariate analysis revealed that educational attainment, knowledge of cervical cancer symptoms, risk factors, prevention, treatment, screening availability, screening procedures, composite knowledge, and attitude towards screening were all significantly associated with cervical cancer screening practices (Table 8). Participants with an associate degree or bachelor’s degree were significantly more likely to exhibit good screening practices (87.5%; *p* = 0.047). Good practice was also significantly associated with adequate knowledge of cervical cancer symptoms (69.6%; *p* = 0.040), risk factors (90.9%; *p* < 0.0001), treatment (91.7%; *p* < 0.001), screening procedures (100%; *p* = 0.002), and composite knowledge (100%; *p* < 0.0001).

Conversely, poor screening practice was significantly associated with poor knowledge of screening availability (100%; *p* < 0.0001) and negative attitudes towards cervical cancer screening (82.9%; *p* < 0.0001).

### 3.11. Independent Factors Associated with Poor Screening Practice

The binary logistic regression analysis identified three independent predictors of poor cervical cancer screening practice: having an associate degree, good knowledge of prevention, and a negative attitude towards screening (Table 9). Surprisingly, participants with an associate degree or good preventive knowledge were less likely to undergo screening. Notably, participants with a negative attitude were significantly more likely to forgo screening (OR = 36.22; *CI* 0.005).

## 4. Discussion

This study examined knowledge, attitudes, cultural beliefs, and cervical cancer screening practices among women in a rural South African community, highlighting the complex interaction of awareness, education, cultural norms, and perceptions of health services that influence screening uptake. Despite relatively high levels of awareness, only just over half of participants reported having ever undergone screening. This disconnect between awareness and practice has also been observed in similar settings where structural, cultural, and psychosocial barriers outweigh knowledge alone in determining health-seeking behavior [23,24,25]. Knowledge levels were generally poor, with only a small proportion of participants demonstrating a comprehensive understanding of symptoms and risk factors. Recognition was largely limited to more obvious symptoms such as vaginal bleeding or foul-smelling discharge, while awareness of HPV as a causative factor was low. These findings are consistent with other studies across Sub-Saharan Africa, where gaps in understanding of cervical cancer risk factors persist despite ongoing public health campaigns [16,26,27]. Symptoms like vaginal bleeding and foul-smelling discharges were widely recognized, but a deeper understanding of risk factors, particularly HPV infection, was limited. This may reflect deficiencies in community-targeted educational strategies and the lack of integration between HPV vaccination programs and cervical cancer awareness [22,28,29,30]. Cultural beliefs were a significant barrier to open discussions about reproductive health, with the majority of participants reporting that sexual organs are not appropriate topics for discussion. Such taboos reflect broader findings from Zimbabwe and Namibia, where patriarchal norms, stigma, and misinformation discourage engagement with preventive services [31,32,33,34]. Our findings share similarities with studies from Zimbabwe and Namibia, where patriarchal norms and cultural taboos also hinder screening uptake; however, the intensity of silence around sexual health discussions in this rural South African context appears particularly pronounced, suggesting a deeper normative constraint. In Nigeria and Ethiopia, low levels of HPV awareness have been reported as primary barriers to screening; however, in our study, awareness was relatively high, yet attitudes and cultural beliefs remained strong determinants of behavior [35,36]. Cultural taboos limit women’s engagement with screening, highlighting the need for community-driven, culturally sensitive interventions such as involving traditional leaders, fostering open dialogue, and using local platforms to reduce stigma and improve uptake. Our findings align with constructs from the Health Belief Model (HBM), for instance, the importance of perceived barriers, benefits, and susceptibility in shaping screening behavior [37]. Complementarily, the Theory of Planned Behavior (TPB) helps conceptualize how attitudes, subjective norms, and perceived behavioral control contribute to intentions and actual screening uptake [38,39]. This finding highlights the need for culturally sensitive interventions that not only provide information but also address misconceptions, stigma, and fatalistic beliefs.

The role of education in influencing screening behavior was notable. Surprisingly, holding a diploma emerged as a predictor of poorer screening practice, contrary to the well-established association between higher educational attainment and improved health-seeking behavior. This counterintuitive finding may reflect contextual factors; women with diplomas in this rural setting could be employed in occupations with inflexible schedules that constrain clinic attendance, or they may experience a misplaced sense of protection stemming from greater health knowledge. The limited number of diploma holders also produced wide confidence intervals, underscoring the imprecision of this estimate. Accordingly, this association should be interpreted with caution and warrants confirmation in a larger, more representative cohort. Women with an associate degree or higher qualifications were more likely to have undergone screening. However, binary logistic regression revealed that holding an associate degree was an independent predictor of poor screening practice. This may be due to the small sample size of associate degree holders and potential confounding factors such as employment type or health service accessibility. Attitudes emerged as a powerful determinant of screening behavior. Participants with negative attitudes were significantly more likely to forgo screening, underscoring the critical role of perceptions, trust in health systems, and fear of diagnosis or pain in shaping health behaviors [40,41,42,43]. Despite the availability of free cervical screening services in South Africa, structural and behavioral barriers persist. Poor knowledge of screening intervals and eligibility criteria further contributes to inconsistent or delayed uptake of screening services. Notably, the WHO’s 90-70-90 strategy for cervical cancer elimination by 2030 emphasizes the need for seventy percent of women to be screened by age 35 and again by 45; however, achieving these targets will be difficult without concerted community-based health education and structural reforms [44,45,46,47]. Our findings indicate that in rural South Africa, cultural beliefs and mistrust, rather than knowledge gaps, are the primary constraints, underscoring the need for context-specific interventions. In addition to barriers, our study identified enablers, including high awareness, recognition of screening as a preventive measure, higher education, and positive attitudes, all of which support uptake and can be leveraged to strengthen interventions. We acknowledge that the wide confidence interval reduces the robustness of this estimate, highlighting the need for cautious interpretation and further investigation into larger samples.

### 4.1. Limitations

This study is not without limitations. First, the use of a convenience sampling method may have introduced selection bias, potentially influenced the distribution of participant characteristics and thereby limiting the representativeness and generalizability of the findings. Second, the reliance on self-reported data may introduce recall and social desirability biases, especially in sensitive topics such as sexual history and health service utilization. Thirdly, the cross-sectional design precludes causal inferences. Lastly, the small sample size and the limited representation of certain educational and age subgroups may affect the robustness of multivariate associations. The wide confidence interval observed in this finding suggests limited precision, which may be due to the small sample size and should therefore be interpreted with caution. We also acknowledged that, as a pilot study, the relatively modest sample size does impose certain limitations, particularly with respect to the precision of effect estimates and generalizability. However, the findings provide valuable preliminary evidence and hypothesis-generating insights that merit further investigation in larger, confirmatory studies. Future studies employing probability-based sampling would provide more robust evidence. Finally, the involvement of medical students as data collectors may have influenced participants’ responses, as their role could inadvertently introduce social desirability bias despite training in neutral data collection techniques.

### 4.2. Future Research

Future research should be directed toward the most critical gaps identified in this study. First, larger and more representative studies are needed to validate the paradoxical association between diploma attainment and poor screening practice, as the small subgroup size in this study limits the robustness of this finding. Second, qualitative and mixed-methods research should be conducted to explore in greater depth the cultural taboos, stigma, and attitudinal barriers that continue to constrain screening uptake, even in contexts where awareness is relatively high. Third, intervention studies are essential to evaluate the effectiveness of culturally tailored, community-driven strategies such as engaging traditional and religious leaders, creating safe spaces for reproductive health dialogue, and integrating cervical cancer screening into routine reproductive health services. Finally, longitudinal designs should be employed to track screening behavior over time and assess the impact of awareness campaigns, the rollout of HPV vaccination, and policy changes on screening uptake. By prioritizing these areas, future work can generate actionable evidence to close the gap between awareness and practice, strengthen screening coverage, and accelerate progress toward the WHO 90–70–90 elimination targets.

## 5. Conclusions

This study demonstrates that while awareness of cervical cancer and screening services is relatively high among women in rural Eastern Cape, actual uptake remains below the WHO 70% target, with only 55.8% ever screened. Negative attitudes, cultural taboos, and mistrust of health services were key barriers, whereas higher education and positive attitudes emerged as important enablers of screening practice. The unexpected association between diploma attainment and poor practice should be viewed as hypothesis-generating, reflecting contextual or sampling factors that require further investigation. Taken together, these findings underscore that improving screening coverage requires more than awareness campaigns. Culturally sensitive, community-driven interventions that actively challenge stigma, engage traditional and religious leaders, and integrate screening into routine reproductive health services are essential to bridging the gap between knowledge and practice. Strengthening these enablers, while addressing structural and cultural barriers, will be critical to accelerating progress toward the WHO 90–70–90 elimination goals in rural and underserved settings.

## Figures and Tables

**Table 1:** Socio-demographic characteristics of the sample population.

Variable	Category	Frequency (n = 95)	Percentage (%)
Age groups	25–35 years	41	43.2
	36–46 years	25	26.3
	47–57 years	18	18.9
	58–68 years	11	11.6
	Median (IQR): 39 (30–48) years; Min–Max: 25–68 years		
Religion	Christianity	90	94.7
	African spirituality	3	3.2
	Zion	2	2.1
Marital status	Single	40	42.1
	Married	39	41.1
	Widowed	6	6.3
	Divorced	3	3.2
	Cohabitation	2	2.1
	Separated	1	1.1
	Decline	4	4.2
Education Status	Secondary school	40	42.1
	Associate degree	16	16.8
	Primary school	16	16.8
	No formal education	12	12.6
	Associate degree /Degree	8	8.4
	Bachelor’s Degree	3	3.2
Occupation	Unemployed	29	30.5
	House wife	25	26.3
	Government	24	25.3
	Self-employed	11	11.6
	Student	6	6.3
Monthly Income	R < 2000	57	60.0
	R > 2000	34	35.8
Sexual Orientation	Heterosexual	94	98.9
	Homosexual	1	1.1

**Table 2:** Reproductive characteristics of study participants residing in Lutubeni.

Variable	Category	Frequency (n = 95)	Percentage (%)
Ever had Sex	Yes	94	98.9
	No	1	1.1
Age of First Sex (Years)	15–18 years	49	51.6
	>18 years	37	38.9
	<15 years	7	7.4
	Median (IQR):18 (16–21) years; Min–Max: 10–30 years		
History of Casual Sex	No	51	53.7
	Yes	43	45.3
	Never had sex	1	1.1
Use of Contraceptive pills or injections	Yes	75	78.9
	No	19	20.0
	Never had sex	1	1.1
Duration of Using Oral Contraceptives	5–9 years	38	40.0
	≥10 years	20	21.1
	<5 years	16	16.8
	Median (IQR): 7 (5–10) years; Min–Max: 1–20 years)		
My mother had cervical cancer	No	93	97.9
	Yes	2	2.1
History of Condom	Yes	72	75.8
	No	22	23.2
	Never had sex	1	1.1
History of Cigarette Smoking	No	83	87.4
	Yes	12	12.6
History of Alcohol Consumption	No	51	53.7
	Yes	44	46.3

**Table 3:** Knowledge level about cervical cancer and screening.

Knowledge of Cervical Cancer	Response n (%), n = 95	
	Yes	No
Knowledge of symptoms of cervical cancer: Poor knowledge 45 (47.4%), Moderate knowledge 27 (28.4%), Good knowledge 23 (24.2%)		
Vaginal bleeding	57 (60%)	38 (40%)
Vaginal foul-smelling discharges	48 (50.5%)	47 (47.9%)
Pain during sex	43 (45.35)	52 (54.7%)
Knowledge related to the risk factor of Cervical cancer: Poor knowledge 58 (61.1%), Moderate knowledge 26 (27.4%), Good knowledge 11 (11.6%)		
Acquiring HPV	23 (24.2%)	72 (75. 8%)
Multiple sex partners	45 (47.4%)	50 (52.6%)
Multi parity	26 (27.4%)	69 (72.6%)
Early sexual intercourse	29 (30.5%)	66 (69.5%)
Long-term oral contraceptive use	30 (31.6%)	65 (68.4%)
Cigarette smoking	39 (41.1%)	56 (58.9%)
Do not know the risk factors	31 (32.6%)	64 (67.4%)
Knowledge related to prevention: Poor knowledge 52 (54.7%), Moderate knowledge 4 (4.2%), Good knowledge 39 (41.1%)		
Vaccination for HPV	38 (40%)	57 (60%)
Avoid multiple sexual partners	61 (64.2%)	34 (35. 8%)
Avoid long-term use of oral contraceptives	40 (42.1%)	55 (57.9%)
Early screening	65 (68.4%)	30 (31.6%)
No smoking	44 (46.3%)	51 (53.7%)
Do not know the prevention	14 (14.7%)	81 (85.3%)
Knowledge related to the treatment of cervical cancer: Poor knowledge 42 (44.2%), Moderate knowledge 41 (43.2%), Good knowledge 12 (12.6%)		
Surgery	68 (71.6%)	27 (28.4%)
Chemotherapy	57 (60%)	38 (40%)
Radiotherapy	15 (15. 8%)	80 (84.2%)
Do not know	20 (21.1%)	75 (78.9%)
Knowledge of cervical cancer screening: Poor knowledge 8 (8.4%), Moderate knowledge 4 (4.2%), Good knowledge 83 (87.4%)		
Is there screening for cervical cancer	86 (90.5%)	9 (9.5%)
Screening service is available at the local clinic	84 (88.4%)	11 (11.6%)
Knowledge related to screening interval: Poor knowledge 66 (69.5%), Moderate knowledge 27 (28.4%), Good knowledge 2 (2.1%)		
Every year	31 (32.6%)	64 (67.4%)
Every three years	22 (23.2%)	73 (76.8%)
Every five years	12 (12.6%)	83 (87.4%)
Do not know	28 (29.5%)	67 (70.5%)
Knowledge related to screening eligibility: Poor knowledge 48 (50.5%), Moderate knowledge 28 (29.5%), Good knowledge 19 (20.0%)		
Women 30 years of age and older	58 (61.1%)	37 (38.9%)
Prostitute	47 (45.5%)	48 (50.5%)
Elderly women	44 (46.3%)	51 (53.7%)
Do not know	21 (22.1%)	74 (77.9%)
Knowledge related to cervical cancer screening procedures		
VIA	1 (1.1%)	94 (98.9%)
PAP smear	84 (88.4%)	11 (11.6%)
Do not know	11 (11.6%)	84 (88.4%)
Composite Knowledge score		
Poor Knowledge		44 (46.3%)
Good knowledge		3 (3.2%)
Moderate knowledge		48 (50.5%)

**Table 4:** Comparison of screening coverage in the study population with WHO 90–70–90 targets.

Age Group (Years)	WHO Target (≥70%)	Study Coverage (%)	Shortfall vs. Target
25–35	70	46.3	−23.7
36–46	70	72.0	+2.0 (met target)
47–57	70	61.1	−8.9
58–68	70	45.5	−24.5

**Table 5:** Attitude of participants from the Lutubeni community towards cervical cancer and screening.

Variables Used to Measure Attitude	SA	A	NADA	DA
	n (%)	n (%)	n (%)	n (%)
Cervical cancer is a highly prevalent disease among women in South Africa	64 (67.4)	20 (21.1)	6 (6.3)	5 (5.3)
Cervical cancer is the leading cause of cancer-related death among women in South Africa	30 (31.6)	38 (40.0)	14 (14.7)	13 (13.7)
Any adult woman, including you, can acquire cervical cancer	35 (36.8)	41 (43.2)	16 (16.8)	3 (3.2)
Cervical cancer cannot be transmitted from one person to another	26 (27.4)	44 (46.3)	13 (13.7)	12 (12.6)
Screening helps in the prevention of cervical cancer	29 (30.5)	48 (50.5)	12 (12.6)	6 (6.3)
Screening causes no harm to the client	30 (31.6)	37 (38.9)	17 (17.9)	11 (11.6)

SA: Strongly Agree; A: Agree; NADA: Neither agree nor disagree; D: Disagree.

**Table 6:** Cultural views regarding cervical cancer and cervical cancer screening.

Variables of Interest	True n (%)	False n (%)
Sexual organs are not a topic for discussion	84 (88.4)	11 (11.6)
A diagnosis with cervical cancer is associated with death	28 (29.5)	67 (70.5)
A diagnosis of cervical cancer means you are having multiple sexual partners	16 (16.8)	79 (83.2)
A diagnosis of cervical cancer means you are promiscuous	18 (18.9)	77 (81.1)
Fear of cervical cancer	21 (22.1)	74 (77.9)
Cervical cancer is perceived to be caused by indecent behavior	31 (32.6)	64 (67.4)
Need approval from the partner for cervical cancer screening	11 (11.6)	84 (88.4)
Cultural views around cervical cancer screening influence my decision to screen	7 (7.4)	88 (92.6)
Traditional medicines are the primary healthcare-seeking option	11 (11.6)	84 (88.4)
Views around cervical cancer screening are the primary reason why I have never been screened for cervical cancer	4 (4.2)	91 (95.8)
One’s sexual organs are private and not supposed to be exposed or touched	87 (91.6)	8 (8.4)

**Table 7:** Cervical screening Practice of the participants.

Variable	Category	Frequency (n = 95)	Percent (%)
Have you ever been screened for cervical cancer	Yes	53	55.8
	No	42	44.2
Where did you screen	Never screened	42	44.2
	Clinic	32	33.7
	Hospital	20	21.1
	Others	1	1.1
How many times did you screen	Once	41	43.2
	Twice	8	8.4
	Thrice	2	2.1
	Four times	1	1.1
	Five times	1	1.1
When was the last time you screened	Last year	28	29.4
	>3 years	12	12.6
	Within the past 3 years	9	9.5
	<3 years	4	4.2
Who initiated you to be screened	Health professional	39	41.1
	Self-initiated	13	13.7
	Others	1	1.1
Have you ever made use of reproductive health services, like HIV or STI testing	Yes	88	92.6
	No	7	7.4

**Table 8:** Factors Associated with Practice Toward Cervical cancer screening.

Associate Factors	Cervical Cancer Screening Practice		X^2^ *p*-Value
	Poor Practice (N = 42)	Good Practice (N = 53)	
Educational status			0.047 *
Bachelor’s Degree	1 (33.3)	2 (66.7)	
Associate degree	3 (18.8)	13 (81.2)	
Associate degree/Degree	1 (12.5)	7 (87.5)	
No formal education	7 (58.3)	5 (41.7)	
Primary school	9 (56.2)	7 (43.8)	
Secondary	21 (52.5)	19 (47.5)	
Knowledge of the symptoms of cervical cancer			0.04
Good	7 (30.4)	16 (69.6)	
Moderate	9 (33.3)	18 (66.7)	
Poor	26 (57.8)	19 (42.2)	
Knowledge related to the risk factor of Cervical cancer			<0.0001 *
Good	1 (9.1)	10 (90.9)	
Moderate	5 (19.2)	21 (80.8)	
Poor	36 (62.1)	22 (37.9)	
Knowledge related to prevention			<0.0001 *
Good	4 (10.3)	35 (89.7)	
Moderate	2 (50.0)	2 (50.0)	
Poor	36 (69.2)	16 (30.8)	
Knowledge related to the treatment of cervical cancer			0.001
Good	1 (8.3)	11 (91.7)	
Moderate	14 (34.1)	27 (65.9)	
Poor	27 (64.3)	15 (35.7)	
Knowledge of screening availability			<0.0001 *
Good	31 (37.3)	52 (62.7)	
Moderate	3 (75.0)	1 (25.0)	
Poor	8 (100.0)	0 (0.0)	
Knowledge related to the Cervical screening procedure			0.002 *
Good	0 (0.0)	1 (100.0)	
Moderate	32 (38.6)	51 (61.4)	
Poor	10 (90.9)	1 (9.1)	
Composite Knowledge			<0.0001 *
Good	0 (0.0)	3 (100.0)	
Moderate	11 (22.9)	37 (77.1)	
Poor	31 (70.5)	13 (29.5)	
Attitude towards cervical cancer and screening			<0.0001
Negative attitude	29 (82.9)	6 (17.1)	
Positive Attitude	13 (21.7)	47 (78.3)	

The “*” indicates the significance of the values of Fisher’s Exact test.

**Table 9:** Independent factors associated with poor screening practice.

Independent Factors Associated with Poor Screening Practice	B	OR (95% CI)
Held an associate degree	−3.278	0.04 (0.002–0.894)
Good knowledge related to prevention	−3.905	0.02 (0.001–0.424)
Negative attitude towards cervical cancer and screening	3.590	36.22 (2.9–453.6)
Model: X^2^ = 85.3; Nagelkerke R^2^ =79.3%; B = Regression coefficient, OR = Odd Ratio		

## Data Availability

The data presented in this study are available on request from the corresponding author due to ethical reasons.

## Abbreviations

The following abbreviations are used in this manuscript:

SSA	Sub-Saharan Africa
OR	Odd Ratio
COBES	Community-Based Education and Service
LMIC	Low- Middle Income Country
HPV	Human papillomavirus
VIA	Visual inspection with acetic acid
HIV	Human immunodeficiency virus
STI	Sexually transmitted infections
HIC	High-income countries
WHO	World Health Organization
hrHPV	High-risk Human papillomavirus
ASRS	Age-standardized relative survival
